# Aging effects on prefrontal cortex oxygenation in a posture-cognition dual-task: an fNIRS pilot study

**DOI:** 10.1186/s11556-018-0209-7

**Published:** 2019-01-11

**Authors:** Uros Marusic, Wolfgang Taube, Shawnda A. Morrison, Lea Biasutti, Bruno Grassi, Kevin De Pauw, Romain Meeusen, Rado Pisot, Jan Ruffieux

**Affiliations:** 1 Institute for Kinesiology Research, Science and Research Centre Koper, Garibaldijeva 1, Koper, Slovenia; 2 Department of Health Sciences, Alma Mater Europaea – ECM, Maribor, Slovenia; 3 Department of Neurosciences and Movement Sciences, University of Fribourg, Fribourg, Switzerland; 4 Department of kinesiology and physiotherapy, Faculty of Health Sciences, University of Primorska, Izola, Slovenia; 5 Department of Medical and Biological Sciences, Udine University, Udine, Italy; 6 Research Group Human Physiology, Vrije Universiteit Brussel, Brussels, Belgium; 7 Faculty of sport, University of Ljubljana, Ljubljana, Slovenia

**Keywords:** Postural control, Balance, Dual-tasking, Executive control, Elderly, Aging, Functional near-infrared spectroscopy (fNIRS), Attention

## Abstract

**Background:**

The aging process alters upright posture and locomotion control from an automatically processed to a more cortically controlled one. The present study investigated a postural-cognitive dual-task paradigm in young and older adults using functional Near-Infrared Spectroscopy (fNIRS).

**Methods:**

Twenty healthy participants (10 older adults 72 ± 3 y, 10 young adults 23 ± 3 y) performed a cognitive (serial subtractions) and a postural task (tandem stance) as single-tasks (ST) and concurrently as a dual-task (DT) while the oxygenation levels of the dorsolateral prefrontal cortex (DLPFC) were measured.

**Results:**

In the cognitive task, young adults performed better than older adults in both conditions (ST and DT) and could further increase the number of correct answers from ST to DT (all *p*s ≤ 0.027) while no change was found for older adults. No significant effects were found for the postural performance. Cerebral oxygenation values (O_2_Hb) increased significantly from baseline to the postural ST (*p* = 0.033), and from baseline to the DT (*p* = 0.031) whereas no changes were found in deoxygenated hemoglobin (HHb). Finally, the perceived exertion differed between all conditions (*p* ≤ 0.003) except for the postural ST and the DT (*p* = 0.204).

**Conclusions:**

There was a general lack of age-related changes except the better cognitive performance under motor-cognitive conditions in young compared to older adults. However, the current results point out that DLPFC is influenced more strongly by postural than cognitive load. Future studies should assess the different modalities of cognitive as well as postural load.

## Introduction

Aging causes a shift from an automatic to a more cortical control of upright posture and locomotion [1–3]. Older adults show increased and more widespread involvement of cortical areas for postural control compared to young adults; notably in the prefrontal cortex [3, 4]. These over-activations in older adults have been interpreted as a dedifferentiation of brain activation, or as a compensation for age-related declines in brain structure and function [3]. This increase in cortical engagement implies that postural processing is more attention-demanding in this age group compared to their younger counterparts. Further evidence for a more conscious, attention-demanding postural control strategy in older age has been provided indirectly by studies using a posture-cognition dual-task paradigm [5, 6].

In everyday life, it is common to experience situations in which a postural task (e.g., standing or walking) is performed concurrently with a secondary task. Older adults are particularly prone to dual-task costs in situations where attentional resources are shared between a postural and one (or more) additional tasks. In fact, it has been shown that the costs of performing a postural and a cognitive task concurrently are greater in older adults [7–10], implying an increased risk of falls. Indeed, the reduced ability to allocate sufficient attentional resources to postural tasks may account for the high number of falls in the elderly [11]. Performance in balance and/or locomotor tasks under dual-task conditions has been shown to be a good predictor of falls [12, 13].

The prefrontal cortex plays a prominent role in postural, cognitive, and dual-task performance. Studies using functional near-infrared spectroscopy (fNIRS) have shown significant activation in the prefrontal cortex, including the dorsolateral prefrontal cortex (DLPFC), during walking [14, 15], in response to a perturbation to upright stance [16], or during performance of a balance task in a semi-immersive virtual reality environment [17, 18]. In the latter two studies, prefrontal oxygenation levels were positively correlated with task difficulty. Higher prefrontal oxygenation levels during standing were found in patients with Parkinsonian syndromes compared to healthy older adults [19]. Thus, it has been suggested that the prefrontal cortex is critical for one’s ability to selectively allocate (visuospatial) attention [15, 16] and to integrate visual and proprioceptive information [18] in order to maintain or regain postural stability. Besides this role in the processing of postural tasks, the DLPFC also plays a key role in working memory tasks [20–22]. Thus, there is evidence that the prefrontal cortex, particularly DLPFC, is crucial for the performance of postural dual-tasks. Studies using dual-task walking paradigms found increases in prefrontal activity when an attention-demanding task was added to normal walking in young adults, while older adults showed smaller increases [14] or even deactivations [23].

To our knowledge, no age-comparative fNIRS study exists to date that has investigated such effects using a standing postural task in combination with a working memory task. Furthermore, in the studies cited above [14, 23], oxygenation levels were measured during walking alone, or during dual-task walking, but not during single-task performance of the additional task. Therefore, the aim of this study was to investigate the effect of performing a standing and a working memory task alone, and concurrently, on prefrontal activity in young and older adults, and to see whether differences in task performance could be explained by changes in prefrontal cortex activity. For this purpose, we measured behavioral performance as well as prefrontal oxygenation levels using fNIRS during standing, counting backwards, and when the two tasks were combined, in both young and older adults.

## Methods

All procedures were carried out in accordance with the ethical standards of the 1964 Declaration of Helsinki and were approved by the National Medical Ethics Committee. Written informed consent was obtained from all participants prior to the study and no payment was provided for participation.

### Participants

Older adults were identified and contacted from the clinical database of an existing EU-funded project entitled “Physical Activity and Nutrition for Quality Ageing” (PANGeA). For the purpose of this study, 10 healthy older adults (for details see Table 1) were randomly selected from a pool of 152 volunteers and recalled to the laboratory for further testing. In addition, 10 healthy young adults (for details see Table 1) were recruited and randomly selected from the database of the study program Applied Kinesiology (University of Primorska, Slovenia). Exclusion criteria included any history or current symptoms of: severe acute metabolic, neuromuscular, and cardiovascular diseases, excessive obesity (over 45% fat), infectious diseases, cancer, bleeding, physical exhaustion, mild cognitive impairment or dementia, critical ischemia of the lower limbs, and patients unable to complete all measurement protocols. All participants were right-handed and had normal or corrected-to-normal vision. All participants indicated on a 5-point Likert scale, where 1 represents a minimum (very bad) and 5 a maximum (very good) score, how much they agreed on their general health status, physical fitness, mental health, and general quality of life. After conclusion of all measurements and a short break, older adults were additionally evaluated with Montreal Cognitive Assessment (MoCA) tool. Characteristics of all participants are presented in Table 1.

**Table 1:** Participants’ characteristics

	Young adults (*N* = 10)	Older adults (N = 10)	*p* value
Sex	7 women	6 women	
Age (years)	22.6 ± 2.8	72.3 ± 3.2	< 0.001
Height (cm)	173.4 ± 6.6	166.8 ± 8.6	0.074
Weight (kg)	75.3 ± 12.2	73.8 ± 11.2	0.798
Education duration (years)	14.3 ± 1.6	14.1 ± 2.1	0.828
MoCA total score		28.0 ± 1.2	
Subjective health assessment:			
a.) General health status	4.3 ± 0.5	3.9 ± 0.6	0.123
b.) Physical fitness	4.2 ± 0.6	4.0 ± 0.8	0.631
c.) Mental health	4.5 ± 0.5	4.2 ± 0.6	0.353
d.) Quality of life	4.4 ± 0.5	4.3 ± 0.7	0.853

*Note:* Data are mean ± SD. MoCA, Montreal Cognitive Assessment

### Study design and experimental tasks

This between-group design study consisted of a dual-task (DT) paradigm that combined a postural task with a cognitive task. The postural task consisted in standing as still as possible in a tandem position while focusing on a black point placed at eye-level approximately one meter in front of the participants. A force plate (AMTI HE600600-2 k, Advanced Mechanical Technology, Inc., Watertown, MA, USA) was used to measure displacements of the center of pressure (COP) in both medio-lateral (m-l) and antero-posterior (a-p) directions. All postural parameters (mean sway path, mean frequency, and mean amplitude) were obtained from the Wise-Coach software (Wise Technologies, Ljubljana, Slovenia). Furthermore, the cognitive task consisted in subtracting serial threes from a randomly chosen number between 400 and 500. Participants were instructed to perform as many correct subtractions as possible while prioritizing correctness over speed and the number of correct answers was counted for each trial.

Upon arrival, all participants received a familiarization period (approximately 10 min), in which they got used to the measuring environment and equipment, as well as the postural and cognitive tasks. Afterwards, the following conditions were performed, during which prefrontal cortex oxygenation was measured: 1) a baseline reference condition (for the fNIRS analysis) that consisted in quiet standing, 2) cognitive single-task (Cognitive ST), 3) postural single-task (Tandem ST), and 4) the cognitive and postural tasks concurrently, i.e., dual-task (DT). The order of the latter two conditions was randomized between participants. Two trials of 60 s and were performed in each condition. All measurements were carried out in a separate and quiet room to avoid any external disturbances. At the end of each trial, participants were asked to subjectively rate their perceived exertion (0–10 Borg scale). Finally, dual-task effects (DTEs) were calculated for all COP parameters and for the cognitive performance following the formula published in [24] and cognitive-motor dual-task interference [25] was further added.

### Cerebrovascular setup

Measurement of prefrontal cortex oxygenation was performed using a single-distance continuous wave fNIRS set-up (Oxymon, Artinis, The Netherlands), using methods described elsewhere [26]. Briefly, this device contained a headset that held a near-infrared emitter with laser light at 780 and 850 nm and a detector pair placed above the DLPFC of the left hemisphere. Optode spacing was 45 mm corresponding to a depth of 20–25 mm. Following instructions by Holtzer and colleagues [14], changes in oxygenated (O_2_Hb) and deoxygenated (HHb) hemoglobin concentrations relative to a 5-s baseline, recorded immediately before the first condition, were calculated for each condition. More specifically, for a given 60 s trial the first and last 10% of the fNIRS data were deleted, meaning each trial had continuous data for 48 s in duration. This was to reduce measurement error at the beginning and end of each trial.

### Statistical analysis

For all parameters, the average of the two trials of each condition was used for further analysis. Data were analyzed with IBM SPSS Statistics 24.0 software for Windows (SPSS, Inc., Chicago, Ill, USA). Homogeneity of variances and normality of the distribution of the parameters was tested with the Levene’s and Shapiro-Wilk’s test, respectively. Two-way mixed-design analyses of variance (ANOVA) with age as a between-subject factor (young vs. older adults) and condition as a within-subject factor (ST vs. DT) was performed on the COP parameters as well as on the number of correct answers for the cognitive task. The hemodynamic variables (O_2_Hb and HHb) were also analyzed using a two-way mixed design ANOVA with the within- and between-subjects factors of age (young vs. older adults) and condition (four levels: Baseline, Cognitive ST, Tandem ST, DT). Significant main or interaction effects were followed up by Bonferroni-corrected post hoc tests. Finally, for the subjective ratings of physical and cognitive load, a Friedman’s ANOVA was used. Statistical significance was set at the level of *p* <  0.05.

## Results

### Baseline characteristics

Table 1 summarizes the participants’ characteristics. The independent sample *t*-test showed that participants varied by age (*p* <  0.001) but were well-matched for all other parameters (*p* ≥ 0.074). Furthermore, older adults showed no signs of cognitive impairments (MoCA score ≥ 26).

### Postural control

Analysis showed no significant main or interaction effects for parameters of COP sway path (total, m-l, and a-p; all *p*s > 0.141).

### Cognitive performance

**Fig. 1:**
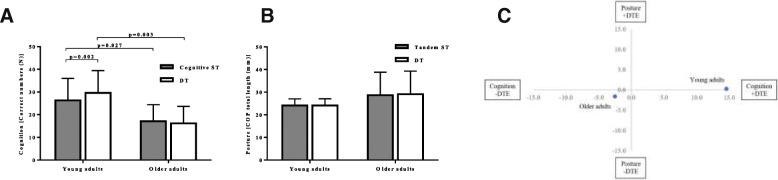
Cognitive performance (serial threes) during Cognitive ST and DT positions (**a**); Total length of body sway in millimeters (**b**); Cognitive-motor dual-task interference (**c**)

A significant interaction effect of age and condition (Cognitive ST vs. DT), *F*(1,18) = 10.655, *p* = 0.004, partial η^2^ = 0.372, was found for the number of correct answers. Young adults gave more correct answers than older adults during the ST (*p* = 0.027) as well as during the DT (*p* = 0.003) condition. Moreover, young adults increased the number of correct answers from the ST to the DT condition (*p* = 0.002), whereas there was no difference between conditions in older adults (see Fig. 1).

### Cognitive-motor dual-task interference

In the cognitive-motor dual-task interference analysis, older adults show a mutual interference, while young adults show mutual facilitation where the majority of improvements were found in cognitive task and almost no change (+ 0.3%) in COP parameters (see Fig. 1 c). Here the only significant change remains the cognitive improvements in young adults from Cognitive ST to DT (*p* = 0.002), and there are no changes observed in the older adults (*p* = 0.404). In the change of COP parameters from Tandem ST to DT, no differences were observed in younger (*p* = 0.756), nor older participants (*p* = 0.112). Moreover, also in the oxygenated hemoglobin no changes from Tandem ST to DT in young (*p* = 0.597), nor old (*p* = 0.752).

### Hemodynamic changes

**Fig. 2:**
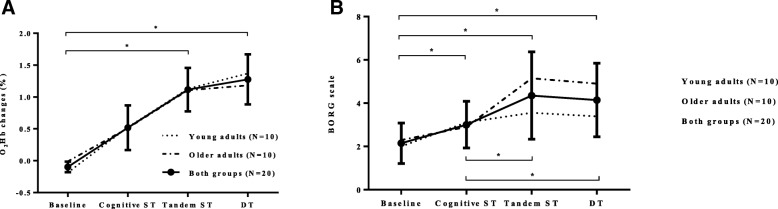
O2Hb changes in all four conditions (**a**); BORG scale of perceived exertion (**b**) *Note:* no significant differences were observed between the two groups. However, significant changes between conditions are marked with *. The calculation of relative O_2_Hb is stated in paragraph “2.3 Cerebrovascular setup”.a.

Hemodynamic changes for O_2_Hb values revealed no significant interaction effect (*p* = 0.898), however there was a significant main effect of condition on O_2_Hb values (see Fig. 2a), *F*(3,54) = 7.329, *p* = 0.002, partial η^2^ = 0.289. Post hoc tests revealed a significant change from Baseline to Tandem ST (*p* = 0.033), and Baseline to DT (*p* = 0.031), while there were no significant changes from Baseline to Cognitive ST (*p* = 0.641) and Tandem ST to DT (*p* = 0.430). Finally, for HHb values, there was no significant interaction (*p* = 0.890), nor main effect of condition (*p* = 0.663).

### Subjective rating of physical and cognitive load

Fig. 2 b depicts Borg values separately for younger and older adults, as well as for both groups together. There was a significant difference in the perceived effort between conditions (χ^2^(3) = 43.006, *p* < 0.001). Post hoc tests were significant between all conditions (*p* ≤ 0.003) except between Tandem ST and DT (*Z* = − 1.271, *p* = 0.204). Additionally, no significant differences were observed between the two age groups for all four conditions for the subjective rating of Borg scale (*p* ≥ 0.063): no differences were obtained for Baseline (*p* = 0.676), Cognitive ST (*p* = 0.507) and Tandem ST (*p* = 0.147), with a non-significant trend for DT (*p* = 0.063)

## Discussion

This study investigated a postural-cognitive dual-task paradigm in young and older adults using fNIRS. The study revealed significant increases in O_2_Hb in the DLPFC when the difficulty of the postural task was increased (i.e., from normal to tandem stance), whereas there were no changes when cognitive load was added to standing. This was true for both age groups with no significant age differences in oxygenation levels. For the behavioral parameters, no significant differences between age groups or conditions were found except for a slightly better performance in the cognitive task from the single- to the dual-task condition in young adults. Finally, a subjective measure of physical and cognitive load revealed no age-related differences and was found to be significantly different between all conditions expect when cognitive load was added to a tandem postural stance.

There were no significant differences observed in DTEs in either the younger or older adults with an exception of improvements observed in the subtracting task in the young adults. When addressing the motor-cognitive dual-task interference, older adults show a mutual interference (slight cognitive as well as postural interference), while young adults show a mutual facilitation (slight postural facilitation and a significant cognitive facilitation) during the most demanding tandem stance condition. The latter could be indicative of higher concentration levels and/or attentional resources recalled during the more demanding postural condition. Accordingly, no differences in prefrontal oxygenation was found between the single- and the dual-task conditions or between age groups. To the contrary, Rosso et al. [27] reported higher activation patterns of the prefrontal cortex and temporal regions in older, compared to young adults. Our results are not directly comparable with theirs due to the fact that they used a different cognitive task (auditory choice reaction time) as well as a different postural task (dynamic stance with feet together and eyes closed). Similar findings than ours were reported in a study that compared walking alone to walking while talking [23]. Other studies using similar paradigms, however, found increased oxygenation levels in the prefrontal cortex during walking while talking compared to normal walking in both young and older adults [14, 15]. The discrepancy between these findings and our results could be explained by differences in the attentional demands of the postural tasks used; possibly, the two-legged standing tasks used in the present study were not challenging enough to induce significant effects. It has been shown in a systematic review [5] that static standing tasks are not always challenging enough to detect age-related changes in dual-task abilities. Indeed, studies using postural tasks with dynamic surface conditions systematically reported greater dual-task costs in older compared to young adults [5]. Similarly, it has been shown that walking is more attention-demanding than standing, in both young and older adults, possibly due to the particular demands of generating bilateral, synchronized limb movements [28]. Further support for this interpretation comes from a study that combined standing tasks with a cognitive task and found increases in DLPFC oxygenation during dual-task only in a one-legged standing condition and not during two-legged standing [29].

Interestingly, one study reported a smaller increase in prefrontal activity from single- to dual-task walking in older compared to young adults [14] while another study even found a deactivation in older adults in one dual-task condition while in young adults there was no change [23]. This might be somewhat surprising since it has been shown that performing postural and cognitive tasks alone increases prefrontal activity more in older than in young adults [3]. It has been suggested that older adults may underutilize the prefrontal cortex when performing an attention-demanding task while walking [14]. This suggestion is supported by two studies [30, 31] showing that transcranial direct current stimulation of the prefrontal cortex improves dual-task performance in older adults. In these studies, the costs of performing a working memory task concurrently with standing or walking were significantly reduced when prefrontal activity was facilitated by the stimulation while it had no influence on single-task performances. Another interpretation is that older adults shift processing resources to other brain areas in dual-task situations [23]. Since in the present study, brain activity was measured only within a limited area of the prefrontal cortex, namely DLPFC, we cannot exclude activity changes in other brain areas in the older adults.

There are some methodological limitations that should be taken into consideration when interpreting the present study. Firstly, the fNIRS system allowed us to perform measurements only within a limited area of the prefrontal cortex, namely DLPFC. Future studies could include a multichannel fNIRS device to cover a larger as well as bilateral brain regions, as similar studies have revealed posture- and age-related interactions in other brain areas, such as the sensorimotor cortex (e.g., Wang et al. [32]). Moreover, a prefrontal hemispheric asymmetry can be found after different cognitive-motor interventions (e.g. an elevation of left ventrolateral PFC activity and a reduction of right ventrolateral PFC activity has been observed in old adults after cognitive training [33] or cognitive-motor training [34]. Secondly, due to the fact that normal aging is also accompanied by declines in executive function, including deficits in processing speed, working memory capacity, and attentional processing (namely in tasks requiring selective and/or divided attention) [35], other cognitive loads (e.g., reciting alternate letters of the alphabet, mental rotation task, serial sevens) aside from serial threes need to be tested. Another limitation goes in the direction of greater differences in age-related discrimination, that would perhaps have been more visible if the variability of COP parameters were available (e.g., standard deviations and/or root mean squares; for useful review see [36]). We further acknowledge that our sample of healthy older adults might not be representative of the general population of older adults. This assumption is supported by the older participants’ subjective ratings of their health and fitness status (see Table 1), which were above the average among other older adults we collected in a larger PANGeA cohort (not published observations). Finally, although small sample sizes between groups are common in the literature, it must be acknowledged here that the matched *n* = 10 samples for each age group represent only the minimum needed for conducting the statistical parameters of the present pilot study (typically between 8 and 10 individuals per homogeneous group).

## Conclusions

In summary, this is the first study to provide an indication that increasing postural demands contributes more to the observed increase in DLPFC activity during a postural dual-task than increasing the cognitive load. However, it should be noted that this finding may also have arisen from the fact that an increase in cognitive demand (i.e., simple mental math: “counting backwards by threes”) may not be directly comparable to the increase in motor demand (i.e., narrowing the base of support: “tandem stance”). The fNIRS measurements showed significantly higher O_2_Hb concentration level changes in the DLPFC when postural task difficulty was increased (from baseline to Tandem ST). In contrast, there were no significant increases in O_2_Hb concentration between the baseline and the dual-task condition (Cognitive ST) nor tandem stance (Tandem ST) and the DT condition. This suggests that changing the posture from normal stance to tandem stance increases cognitive load, at least in the DLPFC, while adding a serial threes cognitive task to tandem stance does not. Understanding the neural contribution to reduced postural control in aging could help developing novel interventions for elderly to reinforce postural control, especially in challenging dual- and multi-task conditions, and improve their quality of life, including a faster return to premorbid daily functioning.
